# Use of Discrete Wavelet Transform to Assess Impedance Fluctuations Obtained from Cellular Micromotion

**DOI:** 10.3390/s20113250

**Published:** 2020-06-07

**Authors:** Tse-Hua Tung, Si-Han Wang, Chun-Chung Huang, Tai-Yuan Su, Chun-Min Lo

**Affiliations:** 1 Department of Biomedical Engineering, National Yang-Ming University, Taipei 11221, Taiwan; d40004001@ym.edu.tw (T.-H.T.); d40404006@ym.edu.tw (S.-H.W.); dk800108@ym.edu.tw (C.-C.H.); 2 Department of Electrical Engineering, Yuan-Ze University, Chung-Li 32003, Taiwan

**Keywords:** ECIS, discrete wavelet transform, cytochalasin B, micromotion, variance, signal magnitude area

## Abstract

Electric cell–substrate impedance sensing (ECIS) is an attractive method for monitoring cell behaviors in tissue culture in real time. The time series impedance fluctuations of the cell-covered electrodes measured by ECIS are the phenomena accompanying cellular micromotion as cells continually rearrange their cell–cell and cell–substrate adhesion sites. Accurate assessment of these fluctuations to extract useful information from raw data is important for both scientific and practical purposes. In this study, we apply discrete wavelet transform (DWT) to analyze the concentration-dependent effect of cytochalasin B on human umbilical vein endothelial cells (HUVECs). The sampling rate of the impedance time series is 1 Hz and each data set consists of 2048 points. Our results demonstrate that, in the Daubechies (db) wavelet family, db1 is the optimal mother wavelet function for DWT-based analysis to assess the effect of cytochalasin B on HUVEC micromotion. By calculating the energy, standard deviation, variance, and signal magnitude area of DWT detail coefficients at level 1, we are able to significantly distinguish cytotoxic concentrations of cytochalasin B as low as 0.1 μM, and in a concentration-dependent manner. Furthermore, DWT-based analysis indicates the possibility to decrease the sampling rate of the micromotion measurement from 1 Hz to 1/16 Hz without decreasing the discerning power. The statistical measures of DWT detail coefficients are effective methods for determining both the sampling rate and the number of individual samples for ECIS-based micromotion assays.

## 1. Introduction

Electrical cell–substrate impedance sensing (ECIS) has existed since 1984 and has been used as a versatile cell-based biosensor to monitor subtle changes in cell-cell and cell-substrate interactions under various experimental conditions [1,2,3,4,5,6,7]. One of the ECIS time course measurements is used to measure the rapid impedance changes in the cell-covered electrodes at a specific frequency with a very high time resolution (sampling rate ≥ 1 Hz). Since electric currents must flow from the electrode surface, underneath the ventral cell surface, and then through the paracellular space, these impedance fluctuations are associated with minute morphological changes in cells and have been referred to as micromotion [2,8]. In addition, they are easily observed in small sensing electrodes, approximately 10^−3^–10^−4^ cm^2^, where the activities of a small population of cells are measured [9]. Cellular micromotion has been studied by ECIS, quartz crystal microbalance (QCM), and surface plasmon resonance (SPR). For example, Tarantola et al. applied ECIS to measure the micromotion of Madin-Darby Canine Kidney (MDCK) cells which were continuously exposed to increasing amounts of colloidal semiconductor quantum dots and gold nanorods and to quantify the in vitro cytotoxicity of these nanoparticles [10]. The transient shape fluctuations of adherent MDCK cells were detected by QCM in real time and the information of cell motility was derived from the noise analysis of the fluctuating resonance frequency [11]. Recently, long-range SPR was used to measure cellular micromotion and the slope of the power spectral density of the optical fluctuations was calculated to determine the micromotion index for the comparison between live and fixed cell layers [12]. In our previous studies, ECIS rapid time collection (RTC) mode was used to measure cellular micromotion in response to temperature change, glucose deprivation, and the exposure of different concentrations of cytochalasin D, cytochalasin B, and carbonyl cyanide 4-(trifluoromethoxy)phenylhydrazone (FCCP) [8,13,14,15]. Impedance time series fluctuations were analyzed by several numerical methods, such as power spectrum, variance, and the Hurst and detrended fluctuation analysis (DFA) exponents, and served as the measures of micromotion. Taken together, these findings demonstrate that the measurement of micromotion may be taken as an indicator regarding cell metabolism, viability, and motility [8,10,11,12,13,14,15].

Despite the success of ECIS-based micromotion measurement, one limitation for this RTC mode is that impedance data are acquired for a specified period of time for each electrode and electrodes are measured one by one. For example, if 2048 data points are taken from the first electrode with one point acquired each second, it will take about 0.57 h and 8.5 h before the second and sixteenth electrodes are measured, respectively. If cells on different electrodes are treated with biological, chemical or physical agents at one time, it is not appropriate to collect data at such different time points. On the other hand, it is also inconvenient to treat cells at different times since the incubator needs to be opened and closed many times during data collection. In ECIS, single-frequency time series (SFT) measurement can be used to follow selected electrodes in turn at the frequency specified. The minimum time will depend upon the number of electrodes being measured as the measurement requires approximately 0.5 s per electrode. Most of this time is taken by adjusting the lock-in amplifier and switching between electrodes. If 16 electrodes are being followed, for example, the minimum time between data points for each electrode will be about 8 s (sampling rate = 1/8 Hz). Although SFT measurement records impedance with a lower time resolution than RTC, it is our objective to analyze RTC data and determine the minimum sampling rate for the data recording of SFT with the same discerning power as RTC.

Cellular responses to their environmental stimulations are usually time dependent. Impedance fluctuations obtained from cellular micromotion, therefore, can be considered as non-stationary biosignals which exhibit temporal changes in spectral features. Some of the techniques used to extract the features from the time–frequency representations of biosignals include short-time Fourier transform (STFT), Hilbert–Huang transform, wavelet transform, etc. [16,17]. The STFT involves multiplying the signal by a short time window to divide the time scale into multiple segments, and applying Fourier transform to each of the windowed segments. The drawback of the STFT approach is the uniform resolution (same duration and bandwidth of the window) for all frequencies, resulting in a tradeoff between the time resolution and the frequency resolution. Wavelet transform, in contrast, uses frequency-dependent windows and provides a way to avoid the problem of fixed resolution caused by STFT.

Discrete wavelet transform (DWT) is a powerful mathematical tool for the analysis of non-stationary biosignals and has been extensively used in the feature extraction of electrocardiogram (ECG), electromyogram (EMG), and electroencephalogram (EEG) signals [18,19]. Previously, a four-level DWT-based multi-level analysis using the Daubechies 4 (dB4) mother wavelet was applied to evaluate the resistance time series measured before and after the electric wound shock of 3T3 cells. The cell powers associated with all four bands of the resistance were calculated as indicators of cellular micromotion [20]. The same DWT-based analysis was also used to demonstrate that the wound healing process of human keratinocyte cells was faster at the negative pressure of 125 mmHg than at ambient pressure [21]. To distinguish human breast cancer cells (MDA-MB-231 and MCF-7) and normal epithelial cells (HaCaT), Das et al. applied a four-level DWT-based multi-level analysis using the Daubechies 8 (db8) mother wavelet to analyze impedance time series measured at 40 kHz and quantify detail coefficients with cellular energy, cellular power, and cellular moments [22]. They reported that cancer cells were shown to have higher cellular energy, cellular power, and cellular moments than normal cells in growth, confluence, and death phases. The fourth level decomposition was selected as a classifier of cell power dissipation, which reflected the work from long-term cellular micromotion. A follow-up study from the same group showed that MDA-MB-231 cells had a higher growth rate and intrinsic resistance to cell death than MCF-7 cells [23]. While the information of the mother wavelet was not mentioned in the follow-up study, there was a discrepancy in these two studies regarding the comparison of cellular energy between MDA-MB-231 and MCF-7 cells [22,23]. 

The concept that entropy analysis can be used to quantify the complexity of biological signals is intriguing [24]. A recent study reports the use of Shannon entropy equation, as a measure of signal unpredictability, to analyze the impedance time series monitored by ECIS and distinguish age-grouped human adipose stem cell (hASC) variability during osteogenic differentiation [25]. Cells from younger donors demonstrate a higher entropy value, indicating that the fluctuations in impedance readings are significant and the collected data points have higher unpredictability which is attributed to higher cell motility and micromotion [25]. In light of this background, we hypothesize that, compared with ECIS-based micromotion data obtained from cells treated with toxins, data of untreated or healthy cells have higher fluctuations, resulting in higher entropy. This study seeks to assess the degree of micromotion by looking at several measures of multi-level DWT detail coefficients. These measures include signal energy, standard deviation (SD), variance (VAR), and signal magnitude area (SMA) and are labeled as Detail-Energy, Detail-SD, Detail-VAR, and Detail-SMA, respectively. Moreover, we downsample the impedance time series and examine the effect of sampling rate on the degree of micromotion. With the knowledge of the minimum sampling rate for sensitively detecting cellular micromotion in response to toxins, the number of individual samples can be determined for the effective use of SFT micromotion measurement in ECIS-based cytotoxicity assays.

## 2. Materials and Methods

### 2.1. Cell Culture

Human umbilical vein endothelial cells (HUVECs; Lonza, Walkersville, MD, USA) were cultured in endothelial cell growth medium (EGM^TM^ BulletKit^TM^, Lonza, Walkersville, MD, USA) under standard conditions of 37 °C and 5% CO_2_. Cells were passaged when they reached 70% confluency and the medium was changed every 2 days thereafter. For ECIS micromotion measurements, HUVECs were inoculated at a density of 10^5^ cells/cm^2^ on the gelatin-coated electrode wells and cultured for 24 h before the addition of cytochalasin B (Sigma, St. Louis, MO, USA). Only cells with less than six passages were used in the experiments.

### 2.2. Measurement of Impedance Time Course by ECIS

**Figure 1:**
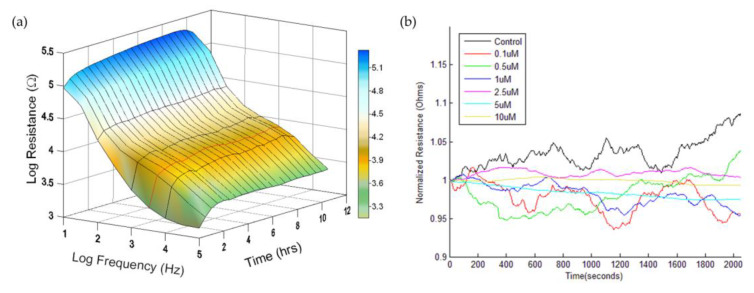
(**a**) Electric cell–substrate impedance sensing (ECIS) measurement of human umbilical vein endothelial cell (HUVEC)attachment and spreading presented as a three-dimensional plot. At time zero, HUVECs were seeded in ECIS electrode wells at cell density of 1E5 cells per cm^2^. The magnitude of the resistance was continually recorded as a function of frequency and experimental time. The red curve represents a time-dependent resistance measured at 4 kHz. (**b**) Normalized resistance time series recorded after 20-h exposure of various concentrations of cytochalasin B or medium only to a confluent HUVECs layer. The final treated concentrations are DMSO as the control (black), 0.1 μM (red), 0.5 μM (green), 1 μM (blue), 2.5 μM (magenta), 5 μM (cyan), and 10 μM (yellow). Each curve has 2048 data points recorded at the sampling rate of 1 Hz. Four independent ECIS experiments were performed (n = 4); in each experiment, two or three independent replicates (electrode wells) were measured for each drug concentration.

The ECIS Z-Theta system, 8W1E electrode arrays, and software for the measurement were obtained from Applied BioPhysics (Troy, NY, USA). Each array consists of eight individual wells, which contain one gold sensing electrode (250 μm diameter) and a much larger gold counter electrode. Please refer to the website of Applied BioPhysics (https://www.biophysics.com/cultureware.php) for detailed information about the 8W1E array configuration. Two 8W1E arrays are used in the ECIS array station for a total of 16 wells per experiment. Each well has a surface area for cell attachment of 0.8 cm^2^ and holds a maximum volume of about 0.5 ml. Cell attachment and the spreading of HUVECs was monitored at 11 different frequencies for at least 24 h after cell inoculation. The confluency of HUVECs was verified electrically by the impedance values. Cytochalasin B in DMSO or DMSO alone as a control was added to each well to give the various final concentrations and the impedance data of all 16 individual wells were measured one after the other. In order to keep the same exposure time, cells were treated with different concentrations of cytochalasin B at different time moments. Following 20 h of cytochalasin B exposure, the rapid time collection (RTC) mode was used to record the time series impedance of the cell-covered electrode at a sampling rate of 1 Hz. An approximate constant AC current of one microampere at the selected frequency, 4 kHz, was applied through the small and the large electrodes, while the voltage was monitored with a lock-in amplifier. Voltage and phase data were converted to resistance or capacitive reactance values. After 2048 data points were continuously acquired from the same electrode, the next electrode was measured. As shown in Figure 1a, the measured resistance at 4 kHz increases drastically after cell attachment and spreading. Since 4 kHz is the selected frequency for RTC measurement, in this study, only the resistance time series data were used for micromotion analysis.

### 2.3. Numerical Analysis of Impedance Fluctuations

We have applied several methods to numerically analyze the impedance fluctuations due to cellular micromotion [8,13,14]. For example, the data set of 2048 points was first split into 64 subsets of 32 points each and the variance (VAR32) was calculated and averaged for all the subsets. The process for calculating the variance (VAR32), the variance of increment (VOI32), and Hurst exponent is the same as we previously described [8]. These methods have been shown to successfully detect the cytotoxic effects of cytochalasin B on HUVECs and 3T3 fibroblasts in a concentration-dependent manner [13,14]. For example, the Hurst exponent can be used as a measure of long-term correlations in the time series. A decline in this exponent has been observed for 3T3 cells 20 h after exposure to cytochalasin B from the lowest concentration to the highest [14]. By comparing these measures with the results analyzed by the other analytical tools we proposed in this study, new algorithms to process the fluctuation data measure by ECIS can be established. These algorithms are described in the following subsection.

#### 2.3.1. Shannon Entropy

As the Hurst exponent is used to quantify the long-term correlations in the fluctuations of the signal [13,14], Shannon entropy can be used as a measure of the signal’s unpredictability [25]. The higher the unpredictability (fluctuations) in the resistance time series, the higher value of the entropy. The Shannon entropy for the resistance time series is calculated by the following Equation (1) [26].

(1) $$H\left(R\right)=-\overset{n}{\underset{t=1}{\sum }}P\left(R_{t}\right)\log_{2}P\left(R_{t}\right)$$

where *H* is entropy, *n*, total time, *P*, probability, *R*, normalized resistance, and *t*, time.

#### 2.3.2. Discrete Wavelet Transform

Discrete wavelet transform (DWT) is a computational algorithm in signal processing that allows us to break down an original time series into different coefficients (components) which describe how the series varies over a broad range of time scales (frequencies). Both detail coefficients (low-scale, high-frequency components) and approximation coefficients (high-scale, low-frequency components) are generated by successively passing a signal through high-pass and low-pass filters. The approximation coefficients from a higher level, after passing through a high-pass and a low-pass filter and down-sampling the signal by a factor of two, generate another set of detail and approximation coefficients at a lower level. This decomposition process is continued until the desired level of decomposition is reached [18,19]. In this study, the DWT method was applied to characterize the impedance fluctuations associated with the micromotion behaviors of HUVECs in response to different concentrations of cytochalasin B. The Daubechies (db) wavelet family, from db1 to db20, was selected as a testing set for the mother wavelet functions. The energy, standard deviation (SD), variance (VAR), and signal magnitude area (SMA) of the detail coefficients at level 1 were calculated by Equations (2)–(4) and labeled as Detail-Energy, Detail-SD, Detail-VAR, and Detail-SMA, respectively.

(2) $$\mathrm{Detail}-\mathrm{Energy}=\sum_{t=1}^{n}cD^{2}\left(t\right)$$

(3) $$\mathrm{Detail}-\mathrm{SD}=\sqrt{\frac{1}{n-1}\sum_{t=1}^{n}{\left(cD\left(t\right)-\bar{cD}\right)}^{2}}=\sqrt{\mathrm{Detail}-\mathrm{VAR}}$$

(4) $$\mathrm{Detail}-\mathrm{SMA}=\frac{1}{T}\sum_{t=1}^{n}\left|cD\left(t\right)\right|$$

These measures of DWT detail coefficients have been used to quantify the degree of cellular micromotion [20,21,22,23]. Essentially, Detail-Energy is a statistic calculated from the detail coefficients. Detail-SD and Detail-VAR measure how far the detail coefficients are spread out from the average. Detail-SMA is a statistical measure of the varying signal magnitude over time [27,28].

### 2.4. Statistical Analysis

Significant differences between the control and each of the experimental groups were observed using Student’s *t*-test. The level of significance was set at * *p* < 0.05, ** *p* < 0.01, and *** *p* < 0.001. All data are expressed as the mean ± standard error.

## 3. Results and Discussion

### 3.1. Real-Time Monitoring of HUVEC Attachment and Micromotions

Cell attachment and the spreading of HUVECs on a gelatin-coated electrode were monitored by ECIS and a typical result was presented as a three-dimensional plot, as shown in Figure 1a. Here, the cells were inoculated at time zero and impedance measurements were performed at 11 different frequencies (62.5 Hz–64 kHz). The frequency-dependent impedance (impedance spectrum) data were continuously recorded for 12 h and the magnitude of the resistance was plotted as a function of frequency and experimental time. The normalized resistance spectra of confluent HUVECs, obtained by dividing the cell-covered resistance as a function of frequency with the corresponding cell-free value, has a peak value at 4 kHz, indicating that the optimal detection frequency is 4 kHz for HUVECs cultured on electrodes 250 μm in diameter [9,13]. In general, confluent HUVECs reach the highest resistance value at about 4 or 5 h after inoculation, as seen from the red curve in Figure 1a, which is the resistance time series measured at 4 kHz. To detect the cellular micromotion of HUVECs 20 h after exposure to different concentrations of cytochalasin B, impedance data were taken every second at 4 kHz for 2048 s. The resistance time series was normalized to the first point of each data set, as shown in Figure 1b, to simply represent the relative changes or fluctuations in resistance, regardless of its overall resistance value. It is noted that, while the use of 8W1E gives a very sensitive measurement, the overall resistance values of HUVECs in response to various concentrations of cytochalasin B display very good reproducibility. From inspecting the normalized resistance curves in Figure 1b, it seems that there is a concentration-dependent decrease in the normalized fluctuations, i.e., a reduction in cellular micromotion. Further statistical analysis of the resistance fluctuations is needed to distinguish the cytotoxic effect of low concentrations of cytochalasin B.

**Figure 2:**
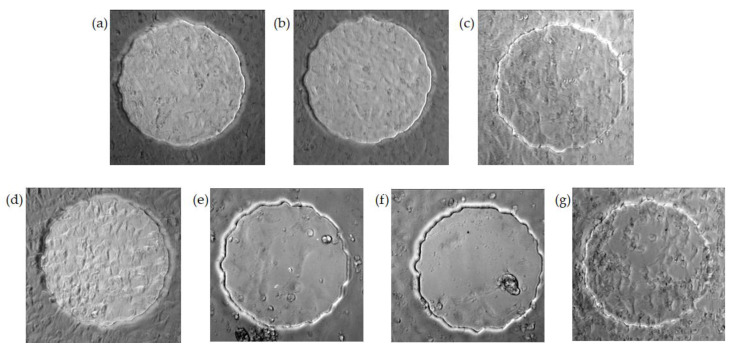
Phase-contrast images of HUVECs on the ECIS 8W1E electrodes taken 20 h after exposure to various concentrations of cytochalasin B. (**a**) DMSO as the control, (**b**) 0.1 μM, (**c**) 0.5 μM, (**d**) 1 μM, (**e**) 2.5 μM, (**f**) 5 μM, and (**g**) 10 μM. The circular area is the sensing electrode, which is 250 μm in diameter.

Cytochalasin B is known to inhibit actin filament polymerization, cell movement, and cytoplasmic division in cultured cells [29,30]. The addition of 10 μM of cytochalasin B to WI38 VA13 cells has been reported to cause an immediate decrease in the resistance measured by ECIS and a large attenuation in the fluctuations [1]. This observation is very intriguing and has stimulated much research in cellular micromotion [8,10,11,12,13,14,15]. We previously demonstrated the effect of cytochalasin B on the overall resistance time course of the HUVEC monolayer. While a rapid drop in resistance was observed after the addition of high concentrations of cytochalasin B (2.5, 5.0, and 10 μM) to cells, it was difficult to tell the difference between the three lowest concentrations (0.1, 0.5, and 1.0 μM) and the control since the changes in the overall resistance were undistinguishable [13]. This result is consistent with the phase contrast images shown in Figure 2, where confluent HUVECs treated with various concentrations of cytochalasin B for 20 h were taken. Visually, the HUVEC monolayer exposed to 0, 0.1, 0.5, and 1 μM cytochalasin B remained confluent, but cells exposed to higher concentrations of cytochalasin B such as 2.5, 5, and 10 μM became rounded and gradually detached from the electrode. One interesting observation is that 0.5 and 1 μM cytochalasin B caused the HUVEC membrane to wrinkle and its surface roughness to increase (Figure 2c,d). This is confirmed in our previous study, showing an increase in HUVEC membrane capacitance in response to a 20-h treatment of 0.5 and 1 μM cytochalasin B [13].

**Figure 3:**
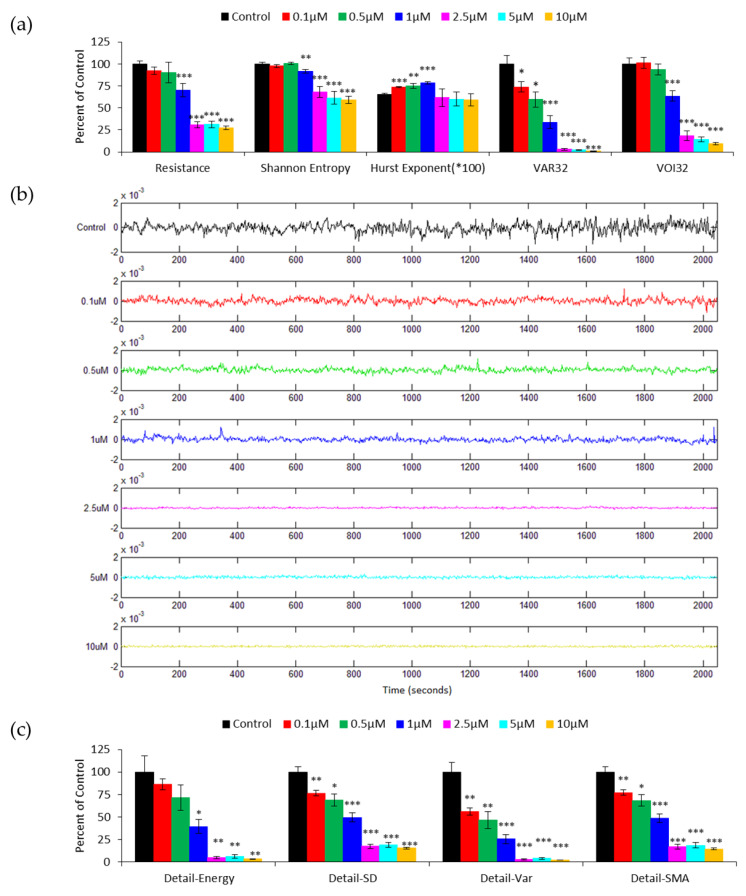
(**a**) Averaged resistance, Shannon entropy, Hurst exponent, variance (VAR32) and variance of increment (VOI32) analysis of the resistance time series shown in Figure 1b. The results are presented as the percentage of control except the Hurst exponent. Here the Hurst exponents are only multiplied by 100 because their original values are in the range of 0.6–0.8, indicating a persistent cell behavior. (**b**) Amplitudes of level 1 discrete wavelet transform (DWT) detail coefficients obtained from the resistance time series shown in Figure 1b. Concentrations of cytochalasin B, from top to bottom, are DMSO as the control (black), 0.1 μM (red), 0.5 μM (green), 1 μM (blue), 2.5 μM (magenta), 5 μM (cyan), and 10 μM (yellow). (**c**) Detail-Energy, Detail-SD, Detail-VAR, and Detail-signal magnitude area (SMA) values of the detail coefficients at level 1. Four independent micromotion experiments were performed (n = 4); in each experiment, two or three replicates (electrode wells) were measured for each concentration. Values shown are mean ± standard error. * *p* < 0.05, ** *p* < 0.01, *** *p* < 0.001. Each value is also expressed as a percentage of control.

To quantify cellular micromotion, we normalized each resistance time series by dividing each data point with their average value and then statistically analyzing the results. Figure 3a shows the results of average resistance, Shannon entropy, Hurst exponent, VAR32, and VOI32 obtained from HUVEC micromotion data for different cytochalasin B concentrations. While the averaged resistance, Shannon entropy, and VOI32 significantly distinguish the four higher concentrations of 1, 2.5, 5, and 10 μM from the lower three, 0, 0.1, and 0.5 μM, VAR32 show a gradual decline in the percentage of control from the lowest concentration to the highest. The Hurst exponent measures the degree of persistence in the time series; however, it cannot distinguish the three highest concentrations from the control.

### 3.2. DWT Analysis of the Effect of Cytochalasin B on HUVEC Micromotion

Figure 3b illustrates the DWT detail coefficients at level 1 for 2048 data points (2048 s) obtained from the resistance time series shown in Figure 1b. Note that after the downsampling, the time resolution is halved and the detail coefficients at level 1 contain only 1024 points. Here, wavelet Daubechies 1 (db1) is chosen as the mother wavelet function. These detail coefficients are features extracted from the time series data at different concentrations of cytochalasin B and distributed along the time axis. Evidently, there is a clear decrease in the fluctuations of the detail coefficients as cytochalasin B concentration increases, and the following numerical analysis confirms this. Figure 3c shows the calculated Detail-Energy, Detail-SD, Detail-VAR, and Detail-SMA, which are measures to quantify the detail coefficients derived from the db1 wavelet, like those shown in Figure 3b. The average value of each measurement at each concentration is calculated from twelve ECIS runs performed in four independent experiments and expressed as a percentage of control. A concentration-dependent decline is generally observed in Detail-SD, Detail-VAR, and Detail-SMA. Furthermore, these three measures are able to distinguish 0.1 μM from the zero concentration (p < 0.01). Although Detail-Energy displays a concentration-dependent trend, it cannot significantly distinguish the differences among the three lowest concentrations, 0, 0.1, and 0.5 μM. This is because the standard errors of the mean of the lowest three concentrations all overlap and their p-values are slightly higher than 0.05. The somewhat big standard error of the Detail-Energy measurement mainly results from the calculation of the square of the detail coefficients, as seen in Equation (2). The results in Figure 3 indicate that signal fluctuations become smaller, i.e., displaying reduced cellular micromotion, in the resistance time series taken at higher concentrations of cytochalasin B. In addition, by looking at the Detail-SD, Detail-VAR, and Detail-SMA values, we are able to detect the effects of the cytochalasin B on HUVECs at concentrations as low as 0.1 μM, the same discernibility as VAR32, as shown in Figure 3a.

**Figure 4:**
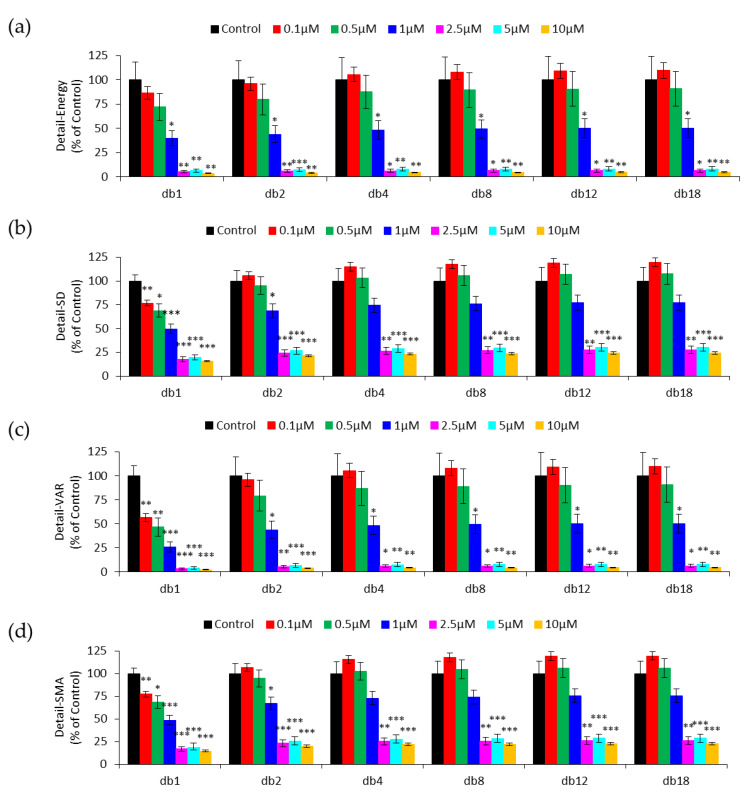
Quantification of detail coefficient from six selected Daubechies mother wavelets by (**a**) Detail-Energy, (**b**) Detail-SD, (**c**) Detail-VAR, and (**d**) Detail-SMA. Four independent micromotion experiments were performed (n = 4); in each experiment two or three replicates (electrode wells) were measured for each concentration. Values shown are mean ± standard error. * *p* < 0.05, ** *p* < 0.01, *** *p* < 0.001. Each value is expressed as a percentage of control.

The selection of an appropriate mother wavelet function is critical in DWT since the characteristics of the transformation and the application of DWT are highly influenced by the choice of the mother wavelet [31]. Different mother wavelet functions applied to the same time series signals may generate different results. Thus, the selection of a proper mother wavelet function is necessary to obtain the optimal performance for the given tasks. In order to determine the most appropriate wavelet in the Daubechies (db) wavelet family (from db1 to db20) for ECIS-based micromotion analysis, detail coefficients derived from each mother wavelet at level 1 were quantified and compared by several measures such as Detail-Energy, Detail-SD, Detail-VAR, and Detail-SMA. Figure 4 shows the four quantitative measures of detail coefficients derived from six selected Daubechies mother wavelets, as 20 data columns in a single graph would be too crowded. For wavelets db2–db20, all four measures cannot significantly distinguish zero concentration from 0.1 and 0.5 μM, and neither can Detail-Energy derived from db1. In contrast, Detail-SD, Detail-VAR, and Detail-SMA using the db1 wavelet successfully distinguish cytochalasin B concentrations as low as 0.1 μM. It is worth noting that Detail-SMA has the smallest standard error of the mean among the three measures, indicating that SMA analysis provides a more sensitive probe.

The db1 wavelet is the same as the Haar wavelet, which is one of the oldest transform functions. As pointed out by Chan and Fu, the Haar (db1) wavelet is an appropriate filter for time series indexing because it allows for a good approximation with a subset of coefficients and can be computed quickly and easily [32]. In this study, the db1 has been found to be the proper mother wavelet in the Daubechies family for dealing with the cytotoxic effect of cytochalasin B on HUVEC micromotion. Previously, db4 and db8 were used for DWT analysis of impedance time series measured by ECIS [20,21,22]. The cells used in these studies include 3T3 fibroblasts, HaCaT keratinocyte cells, and two types of human breast cancer cells, MCF-7 and MDA-MB-231. Since this is an uncharted territory, we suppose that, for different cell lines, cell culture conditions, or sampling rates, the selection of a proper mother wavelet function is still needed. However, it is time-consuming to compare the results of different mother wavelet functions applied to a set of time series data obtained by ECIS and then select the optimal mother wavelet function for optimal performance in wavelet signal processing. We are developing a selection algorithm which can determine the proper mother wavelet function for processing ECIS-based time series signals. We hope this can open the door to further applications of DWT in the feature extraction of ECIS data, with measures of DWT detail coefficients for different cell types and under various culture environments. There are several compressive sensing algorithms that need certain learning methods to find the proper basis/parameters for signal transform processing. One such example is the Bayesian learning method which alternatively and iteratively finds the parameters in a known model function to influence the reconstruction performance [33]. The Bayesian learning method can also be applied in DWT to find the best mother wavelet function. Since the processing procedure for analyzing ECIS-based time series data is still under development, any accessible and efficient method is worth trying in order to find the proper mother wavelet function.

### 3.3. Effect of Downsampling on Micromotion Analysis

**Figure 5:**
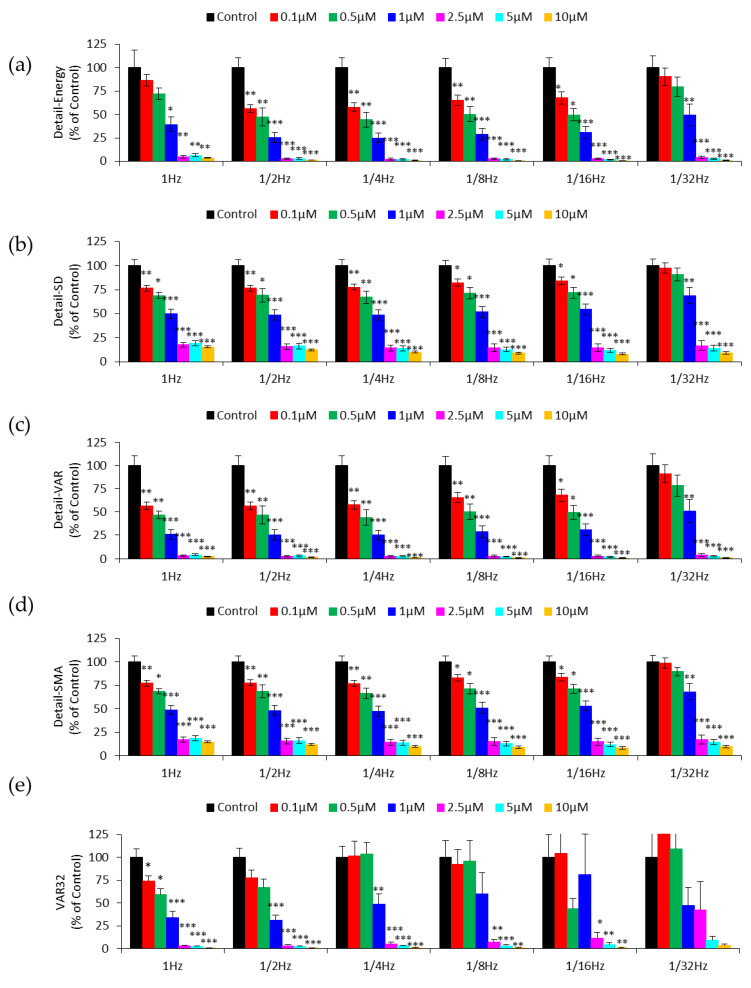
Numerical analysis of the resistance time series after successive downsampling from 1 Hz to 1/32 Hz by (**a**) Detail-Energy (**b**) Detail-SD (**c**) Detail-VAR (**d**) Detail-SMA (**e**) VAR32. Values shown are mean ± standard error. * *p* < 0.05, ** *p* < 0.01, *** *p* < 0.001. Each value is expressed as a percentage of control.

Using the original impedance time series taken at the sampling rate of 1 Hz for 2048 points, we can resample the data as if they were taken at a sampling rate of 1/2 Hz for 1024 points. The downsampling process is repeated for sampling rates of 1/4, 1/8, 1/16, and 1/32 Hz, and the data sets are characterized by DWT-based analysis. One purpose of downsampling data in this way is to make predictions about the effect of sampling rate on micromotion analysis. Figure 5a–d summarizes the Detail-Energy, Detail-SD, Detail-VAR, and Detail-SMA values of the detail coefficients at level 1 for the original and downsampled data. The first thing we notice in Figure 5b–d is that Detail-SD, Detail-VAR, and Detail-SMA values obtained from data sampled from 1 to 1/16 Hz can distinguish cytochalasin B levels as low as 0.1 μM in a concentration-dependent manner (p < 0.05). Furthermore, all four measures can only distinguish cytochalasin B levels as low as 1 μM after downsampling the resistance time series to 1/32 Hz (p < 0.05). The result indicates that these three measures provide a sensitive assessment, across a range of sampling rates, from 1 to 1/16 Hz, of the cytotoxic effect of cytochalasin B on HUVECs. Interestingly, at the 1 Hz sampling rate, Detail-Energy can only distinguish the concentrations as low as 1 μM from the control. However, as shown in Figure 5a, it can distinguish 0.1 μM from the control (p < 0.05) after downsampling data from 1 Hz to 1/2, 1/4, 1/8, and 1/16 Hz. This result is intriguing and suggests that the energy or power of the detail coefficients at different levels of DWT-based analysis may have different detection sensitivities of cellular micromotion [20,21,22,23].

The calculated VAR32 values of the same downsampled data are shown in Figure 5e. In comparison with the results in Figure 5a–d, VAR32 measures distinguish cytochalasin B levels as low as 1 μM at sampling rates of 1/2 and 1/4 Hz, and 2.5 μM at 1/8 and 1/16 Hz. One possible reason to account for this less sensitive outcome is that downsampling by two doubles the time scale of the 32 data points for calculating VAR32. If the 32 s time scale is maintained, VAR16 should be calculated for data series sampled at the 1/2 Hz sampling rate, VAR8 for 1/4 Hz, etc. In this way, VAR16 at 1/2 Hz and VAR8 at 1/4 Hz are as good as VAR32 at the 1 Hz sampling rate (data not shown). Downsampling can also be used to provide an additional insight into the time series data being studied and identify the minimum sampling rate requirement for data collection. According to the Nyquist sampling theorem, the sampling rate should be at least two times as fast as the signal’s highest frequency component. The sampling rate of the single-frequency time series (SFT) mode in ECIS is about 1/8 Hz, two times as fast as 1/16 Hz for consecutively measuring 16 electrode wells, indicating the possible use of SFT measurement in ECIS-based micromotion assay. With this information, the impedance data of electrode wells recorded by SFT measurement will be confidently used with the same discerning power as RTC.

DWT has been applied in biomedical signal processing for years, but its application in impedance-based cellular assays remains to be developed and clarified. Detail coefficients, often considered as signal noise, are eliminated or reduced to give clear signal contours in many cases. However, the noise in the measured impedance time series is usually associated with cellular activities. In this study, we apply DWT to analyze the impedance fluctuations obtained from cellular micromotion. we use only one level of DWT decomposition instead of multi-level decomposition in order to compare it with the variance methods (VAR32, and VOI32.). Because of the use of the db1 mother wavelet, if we downsample resistance time series data by two, four and eight, the detail coefficients at level 1 will be, respectively, equivalent to the level 2, 3, and 4 detail coefficients obtained from the original time series. From looking at measures such as Detail-SD, Detail-VAR, and Detail-SMA, at sampling rate from 1 to 1/16 Hz, we are able to detect the effect of cytochalasin B at concentrations as low as 0.1 μM. Downsampling the resistance time series from the 1 Hz to the 1/16 Hz sampling rate also indicates that four-level DWT-based multiscale analysis can be used to analyze an impedance time series obtained from ECIS-based micromotion assays.

It is our ultimate goal to develop and test more analytical tools for ECIS-based micromotion assays, including RTC and SFT measurements, with which users are able to investigate cell motility and viability in vitro under various environmental conditions. Our future work will focus on the application of DWT-based multi-level analysis in the feature extraction of the impedance time series measured by ECIS. In addition, we will develop a selection algorithm to determine the proper mother wavelet function for ECIS-based signal processing.

## 4. Conclusions

In this study, ECIS is applied to monitor impedance time series of HUVECs in response to different concentrations of cytochalasin B. The impedance fluctuations due to cellular micromotion are quantified by several measures of DWT detail coefficients at level 1. These measures include Detail-Energy, Detail-SD, Detail-VAR, and Detail-SMA. Our results demonstrate that, in the Daubechies (db) wavelet family, db1 is the optimal mother wavelet function for DWT-based analysis to assess the effect of cytochalasin B on HUVECs. In addition, Detail-SD, Detail-VAR, and Detail-SMA can distinguish cytochalasin B concentrations as low as 0.1 μM across a sampling rate range from 1 to 1/16 Hz. We believe this is the first study to apply DWT-based analysis to investigate the bioimpedance time series caused by cellular micromotion, measured using the RTC mode at a sampling rate of 1 Hz. With the information of the sampling rate requirement for detecting cellular micromotion, the number of individual samples can be determined for the effective use of SFT micromotion measurements. The DWT-based analysis applied in this study opens the door to assessing impedance fluctuations and exploring cell behaviors in response to environmental stimuli using ECIS-based methods.
